# *Extract of a Letter from the Reverend* Charles Perceval *to* Robert Perceval, *M. D. and M. R. I. A.*

**Published:** 1794

**Authors:** 


XV. *Extract of a Letter from the Reverend* Charles Perceval *to* Robert Perceval, *M. D. and M. R. I. A.* Vide *Transactions of the Royal Irish Academy.* Vol. IV. 4to. Dublin.

THIS letter contains an account of Jane Bern, a girl, eleven years old, low in ſtature, but of an healthy appearance, whom Mr. Perceval accidentally met with in the charter ſchool at Dunkerrin, in the King's County, and whoſe eyes are conſtructed in an extraordinary manner.

Their motion, inſtead of a regular horizontal one, from left to right, and *vice verſa*, is tremulous in all directions, and partly perpendicular, with a prominent motion of the globe of the eye; what lateral motion the eye is capable of is ſhort, interrupted, and gives that organ the appearance of being bound by ligaments, from which it ſtruggles to get free. The child cannot eaſily look upwards, or ſee any object placed above her eyes; and when ſhe reads, which ſhe does without any heſitation tion or difficulty, ſhe reads perpendicularly from the bottom upwards, and holds the book accordingly.

The whole globe of the eye is of a reddiſh caſt, the white is ſtreaked with ſtriæ of a fainter red; the iris is of an uniform deep red, approaching to brown; both her eyes are weak and watery: and, when turned from the light, glow with a more fiery and vivid colour than when expoſed to it.

Mr. Perceval obſerves, that although ſhe reads very well, and ſeems poſſeſſed of a moderate plain underſtanding, ſhe is unable to write, knit, or ſpin. She has remarkably fine hair, it ſeems, of the colour of flax, but conſiderably whiter.

The miſtreſs of the ſchool informed him that the child was ſent to her from a nurſery in the county of Longford, in the North of Ireland.

Mr. Perceval was not informed whether any more of her family had theſe peculiarities.

The ſingularity of this caſe ſeems to conſiſt in the motion of the eyes; for the redneſs of the iris, and the white colour of the hair, evidently ſhow that it muſt be claſſed with thoſe inſtances inftances of lufus naturæ *, which depend on a white colour of the pigmentum of the eye, correfponding with the colour of the hair, and which have been fo ably defcribed by the late Mr. John Hunter; and likewife by Dr. Blumenbach †.

* For the application of this term here we have the authority of Mr. Hunter, who, in his Effay on the Colour of the Pigmentum of the Eye in different Animals, obferves that "the variation of colour appears moft remark-"able when a white ftarts up, either where the whole fpe-"cies is black, as in the crow or blackbird; or where only "a certain part of the fpecies is black (but permanently "fo), as a white child born of black parents; and that a "perfectly white child, whofe hair is white, and who has "the pigmentum alfo white, though born of parents who "are fair, fhould as much be confidered as a play of Na-"ture as the others. All thofe lufus naturæ," he adds, "fuch as the white negro, the pure white child of fair "parents, the white crow, the white blackbird, white "mice, &c. have likewife a white pigmentum correfpond-"ing with the colour of the hair, feathers, and fkin." See Obfervations on certain Parts of the Animal Oeconomy. By John Hunter. 4to, London, 1786, page 204. Editor.

† *Vide* Jo. Frid. Blumenbachii de Oculis Leucæthiopum et Iridis motu Commentat. 4to. Goettingæ, 1786.

